# Medieval Wisdom and Modern Diagnosis: A 9th-Century Physician’s OCD Description vs. ICD–11

**DOI:** 10.1192/bjo.2025.10187

**Published:** 2025-06-20

**Authors:** Usman Abdul-Quayum, Suleman Jehanger, Imran Ali

**Affiliations:** ^1^NHS Greater Glasgow & Clyde, Glasgow, United Kingdom; ^2^Priory Hospital, Preston, United Kingdom

## Abstract

**Aims:** This study explores the historical roots of Obsessive–Compulsive Disorder (OCD) through an analysis of a ninth-century treatise *Sustenance of the Soul* by Abu Zayd al-Balkhi. It aims to compare al-Balkhi’s descriptions and treatments of OCD with the modern-day classification and understanding of ICD–11. We hypothesise that al-Balkhi could offer a more nuanced, holistic understanding of OCD, including recognition of its physical, psychological, and social aspects, and that this historical perspective can offer valuable insights into current psychiatric practices.

Abu Zayd al-Balkhi, a notable Muslim polymath of the ninth-century, authored *Sustenance of the Soul*, a treatise addressing mental health and wellbeing. Al-Balkhi connected mental and physical health, describing psychological issues that resemble what we now classify as OCD. His work anticipated aspects of modern therapeutic approaches, such as cognitive-behavioural techniques, social support, and activity engagement, to manage obsessive-compulsive symptoms. This historical analysis seeks to illustrate the relevance of early descriptions of mental health disorders in shaping current diagnostic and treatment approaches.

**Methods:** A reading of the text took place in 3 stages, firstly to gain an overview of the key themes in the chapter, secondly to extract key psychological terms being explored and their understanding and lastly to compare and contrast with sections in the ICD–11 classification. The analysis explored themes such as symptomatology, aetiology, and therapeutic techniques, mapping these historical perspectives to contemporary understandings of OCD. The process was checked with another researcher.

**Results:** Al-Balkhi identified obsessional behaviours as stemming from both psychological and physical factors, whilst also suggesting that OCD has a strong heritable component. He recognised that obsessive thoughts could harm physical health and that sufferers might experience cognitive distortions, such as catastrophic thinking – an approach now central to cognitive-behavioural therapy (CBT). Al-Balkhi also noted the aggravation of symptoms through isolation and inactivity, highlighting social and behavioural strategies to mitigate symptoms, which resonates with modern therapeutic recommendations.

**Conclusion:** This study reveals that al-Balkhi’s observations on OCD correlates to a significant degree with the ICD–11 classification. His writings integrated social, psychological, and physical treatments, which is argued to be advanced for his time. His insights offer a framework that complements and deepens current understandings of OCD, underscoring the potential benefits of examining historical perspectives in psychiatry. Al-Balkhi’s work demonstrates the value of integrating historical knowledge into modern practice, promoting a more holistic approach to treating psychiatric disorders.

